# TREM-1 expression on neutrophils and monocytes of septic patients: relation to the underlying infection and the implicated pathogen

**DOI:** 10.1186/1471-2334-11-309

**Published:** 2011-11-04

**Authors:** Thekla Poukoulidou, Aikaterini Spyridaki, Ira Mihailidou, Petros Kopterides, Aikaterini Pistiki, Zoi Alexiou, Michael Chrisofos, Ioanna Dimopoulou, Panagiotis Drimoussis, Evangelos J Giamarellos-Bourboulis, Ioannis Koutelidakis, Androniki Marioli, Anna Mega, Stylianos E Orfanos, Maria Theodorakopoulou, Christos Tsironis, Nina Maggina, Vlassios Polychronopoulos, Iraklis Tsangaris

**Affiliations:** 1 4th Department of Internal Medicine, University of Athens, Medical School, ATTIKON General Hospital, 1 Rimini Str., 12462 Athens, Greece; 2 2nd Department of Critical Care, University of Athens, Medical School, ATTIKON General Hospital, 1 Rimini Str., 12462 Athens, Greece; 3 1st Department of Internal Medicine, "Thriasion" Elefsina General Hospital, Leoforos Gennimata, 19600 Magoula, Greece; 4 2nd Department of Urology, "Sismanogleion" Athens Hospital, 1 Sismanogleiou Str., 15126 Maroussi, Greece; 5 Intensive Care Unit, "Ippokrateion" Athens General Hospital, 114 Vassilis Sofias Str., 11527 Athens, Greece; 6 2nd Department of Surgery, University of Thessaloniki, Medical School, 41 Ethnikis Aminis Str., 54635 Thessaloniki, Greece; 7 2nd Department of Internal Medicine, "Sismanogleion" Athens Hospital, 1 Sismanogleiou Str., 15126 Maroussi, Greece; 8 Intensive Care Unit, "Laikon" Athens General Hospital, 17 Aghiou Thoma Str., 11527 Athens, Greece; 9 Department of Plastic Surgery, 251 Air Force General Hospital, 3 Kanellopoulou Str, 112527 Athens, Greece; 10 Intensive Care Unit, "Aghia Olga" Athens General Hospital, 3-5 Aghia Olga Str., 14233 Nea Ionia, Greece; 11 3rd Department of Pulmonary Medicine, "Sismanoglion" Athens Hospital, 1 Sismanogleiou Str., 15126 Maroussi, Greece

## Abstract

**Background:**

Current knowledge on the exact ligand causing expression of TREM-1 on neutrophils and monocytes is limited. The present study aimed at the role of underlying infection and of the causative pathogen in the expression of TREM-1 in sepsis.

**Methods:**

Peripheral venous blood was sampled from 125 patients with sepsis and 88 with severe sepsis/septic shock. The causative pathogen was isolated in 91 patients. Patients were suffering from acute pyelonephritis, community-acquired pneumonia (CAP), intra-abdominal infections (IAIs), primary bacteremia and ventilator-associated pneumonia or hospital-acquired pneumonia (VAP/HAP). Blood monocytes and neutrophils were isolated. Flow cytometry was used to estimate the TREM-1 expression from septic patients.

**Results:**

Within patients bearing intrabdominal infections, expression of TREM-1 was significantly lower on neutrophils and on monocytes at severe sepsis/shock than at sepsis. That was also the case for severe sepsis/shock developed in the field of VAP/HAP. Among patients who suffered infections by Gram-negative community-acquired pathogens or among patients who suffered polymicrobial infections, expression of TREM-1 on monocytes was significantly lower at the stage of severe sepsis/shock than at the stage of sepsis.

**Conclusions:**

Decrease of the expression of TREM-1 on the membrane of monocytes and neutrophils upon transition from sepsis to severe sepsis/septic shock depends on the underlying type of infection and the causative pathogen.

## Background

Septic syndrome is one of the leading causes of death. Its great lethality had led to several randomized trials of the administration of various types of immunotherapy. The concept of all these types of therapeutic approach was to modulate the exaggerated immune response of the host [1]. However most of results were disappointing. Several probable explanations for these failures have been given; among them the heterogeneity of patients is the most likely. This probably has to do with the type of underlying infection, the causative microorganism and the co-morbid conditions. In a recent prospective study of the Hellenic Sepsis Study Group http://www.sepsis.gr 505 patients were enrolled; changes of the innate and adaptive immunity were evaluated with immunophenotyping performed within the first 24 hours from diagnosis. Results showed that changes occurring during transition from sepsis to severe sepsis or septic shock differed in relation with the type of underlying infection [2].

Triggering receptor expressed on myeloid cells -1 (TREM-1) is a pattern-recognition receptor expressed on neutrophils and monocytes. It participates in innate immune responses and it is activated in the event of disseminated bacterial infections like sepsis. Expression of TREM-1 is up-regulated after stimulation with bacterial and fungal products. This leads to production of pro-inflammatory mediators, mainly of tumour necrosis factor-alpha and of interleukin-8. Although the exact ligand of TREM-1 remains unknown, it is believed that microbial molecules like endotoxins of the cell wall of Gram-negative bacteria and peptidoglycan of the cell wall of Gram-positive cocci may stimulate TREM-1 [3-9]. Missing identification of the exact ligand for TREM-1 creates the hypothesis that TREM-1 expression on neutrophils and monocytes in sepsis may differ according to the type of infection causing sepsis and/or the type of implicated bacteria.

Based on recent data for the crucial role of the type of the underlying infection in immune responses in sepsis [2], the purpose of the present study was to investigate the pattern of TREM-1 expression on cells of myeloid origin in a prospective cohort of sepsis in relation with the causative infection and the offending pathogen.

## Methods

### Study design

This is a prospective, multicentre clinical study conducted over the period June 2009-December 2009. None of the patients was enrolled in the studies already conducted and published by the Hellenic Sepsis Study Group [2,10,11]. The protocol was approved by the Ethics committees of all hospitals of the participating study sites. Written consent was provided from patients or their first-degree relatives for patients unable to consent.

Inclusion criteria were: a) age ≥ 18 years; b) sepsis due to either lower respiratory tract infection or acute pyelonephritis or intrabdominal infection or primary bacteremia/fungemia; and c) blood sampling within less than 24 hours from advent of signs of sepsis.

Exclusion criteria were: a) HIV infection and b) neutropenia defined as an absolute neutrophil count lower than 1000 neutrophils/mm^3^.

Patients were classified as uncomplicated sepsis and as severe sepsis and/or septic shock according to the criteria of the ACCP/SCCM [11,12].

Acute infection of the lower respiratory tract was defined by all the following [13]: (1) physical signs compatible with a lower respiratory tract infection; and b) new pulmonary infiltrates on chest x-ray. It was divided into community-acquired pneumonia (CAP) if the patient had no history of contact with any hospital environment within the last 3 months; and hospital-acquired pneumonia (HAP) if either the patient had some contact with hospital environment within the last 3 months or infection was diagnosed more than 48 hours after hospital admission without being under incubation upon admission.

Ventilator-associated pneumonia (VAP) was HAP presented when a patient under intratracheal intubation and mechanical ventilation for ≥ 48 hours had all the following [14,15]: a) core temperature > 38°C or < 36°C; b) purulent tracheobronchial secretions; and c) new pulmonary infiltrates on chest x-ray.

Acute pyelonephritis was defined for every patient with all the following [16]: a) core temperature > 38°C; b) radiological evidence consistent with the diagnosis; and c) ≥ 10 white blood cells in centrifuged urine sample or ≥ 2 + in urine stick for white blood cells and nitrite.

Acute intra-abdominal infection was defined for every patient with all the following [17] a) core temperature > 38°C; and b) radiological evidence (abdominal x-ray, abdominal ultrasound, abdominal or computed tomography) consistent with an acute abdominal infection.

Primary bacteremia or fungemia was defined for any patient with all the following [17]: a) peripheral blood culture positive for Gram-positive or Gram-negative bacteria or fungal species. Coagulase-negative *Staphylococcus *spp and skin commensals were considered contaminants unless isolated at least two times or isolated from both a peripheral vein and a central catheter and they had the same antibiograms; and b) absence of any primary site of infection after extensive patient work-out.

### Patients' follow up

Seven ml of blood were sampled after venipuncture of one forearm vein under aseptic conditions; five ml were collected into a heparin-coated tube for flow cytometry; another two ml were collected into pyrogen-free tubes. Tubes were transported within one hour via a courier service to the central lab located at the 4^th ^Department of Internal Medicine, ATTIKON General Hospital.

Enrolled patients were followed-up on a daily basis for a total of 28 days. APACHE II scores were calculated upon enrolment.

### Flow cytometry

Red blood cells were lysed with ammonium chloride 1.0 mM. White blood cells were washed three times with PBS (pH. 7.2) (Merck, Darmstadt, Germany) and stained with the monoclonal antibody anti-TREM-1(R&D Systems, Minneapolis, USA) at the fluorochrome phycoerythrin (PE, emission 575 nm). The incubation took place at 4°C for 45 min in the dark. After reconstitution with 0.5 ml phosphate buffered saline pH: 7.2 cells were analyzed through the EPICS XL/MSL flow cytometer (Beckman Coulter Co, Miami, FL, USA) with gating for neutrophils and monocytes based on their characteristic forward and side scattering. IgG isotypic negative controls PE conjugated (IgG1) were applied before the start of analysis. Results were expressed as % of gated cells and as mean fluorescence intensity (MFI).

### sTREM-1 measurements

Pyrogen-free tubes were centrifuged and serum was kept refrigerated at -70°C until assayed. Concentrations of soluble TREM-1 (sTREM-1) were measured in duplicate by an enzyme immunoassay (R&D Inc, Minneapolis, USA). The lower detection limit was 15.1 pg/ml.

### Statistical analysis

Results were expressed as means ± SE. Comparisons of baseline qualitative characteristics were done by X^2^-test and of quantitative baseline characteristics by the Student's t-test. Comparisons of TREM-1 expression between patients at sepsis and patients at severe sepsis/shock with the same type of infection or pathogen were done by the Kruskall-Wallis test; those between different types of infections or pathogens separately for patients at sepsis and patients at severe sepsis/shock were done by the Mann-Whitney U test. Correlation between APACHE II score and TREM-1 expression was done according to Spearman's rank of order. P values below 0.05 after adjustment for multiple comparisons were considered significant.

## Results

A total of 213 patients were enrolled in the study; 125 with sepsis and 88 with severe sepsis/shock. Their demographic and clinical characteristics are shown in Table 1. As expected, APACHE II scores and mortality were greater among patients with severe sepsis/shock.

**Table 1:** Demographic and clinical characteristics of patients enrolled in the study.

	Sepsis	Severe sepsis/shock	p
Number	125	88	
Male/Female	69/56	43/45	0.307
Age (years, mean ± SD)	62.5 ± 22.2	72.5 ± 13.1	< 0.0001
APACHE II score	11.52 ± 5.76	22.68 ± 8.50	< 0.0001

Type of infection (n)			p: 0.004
Acute pyelonephritis	56	18	
Pneumonia (CAP)	16	20	
Intra-abdominal Infection	30	20	
Bacteremia	13	15	
VAP/HAP	10	15	

Predisposing Factors (n, %)			0.188
Chronic Renal Disease	15 (12.0)	12 (13.6)	
Chronic Obstructive Pulmonary Disease	10 (8.0)	10 (11.4)	
Heart Failure	9 (7.2)	17 (19.3)	
Diabetes mellitus type 2	27 (21.6)	17 (19.3)	
Solid tumor malignancy	5 (4.0)	7 (7.9)	

Isolated pathogens irrespective of source (n, %)			0.037
*Escherichia coli*	21 (16.8)	4 (4.5)	
*Pseudomonas aeruginosa*	7 (5.6)	5 (5.7)	
*Klebsiella pneumoniae*	5 (4)	9 (10.2)	
*Acinetobacter baumannii*	2 (1.6)	7 (7.9)	
Other Gram-negatives	10 (8.0)	4 (4.5)	
*Staphylococcus aureus*	2 (1.6)	2 (2.3)	
Other Gram-positive	2 (1.6)	2 (2.3)	

Death	10 (8.0)	49 (55.7)	< 0.0001

**Figure 1:**
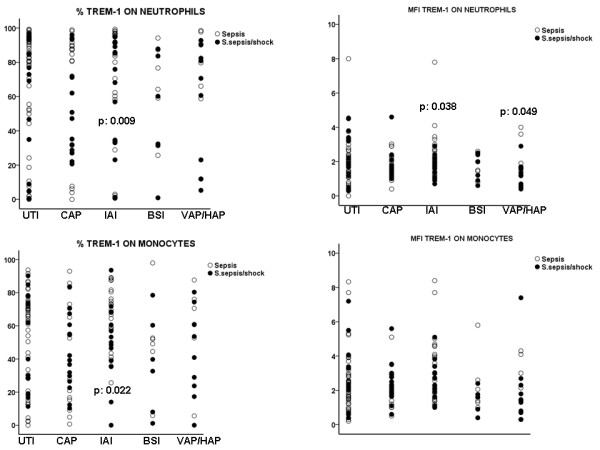
**Expression of TREM-1 on the first day of presentation of sepsis and of severe sepsis/septic shock on the surface of neutrophils and monocytes related with the causative type of infection**. P values indicate statistically significant differences between sepsis and severe sepsis/shock at the specific infection.

Expression of TREM-1 on the membrane of neutrophils and monocytes of the first day of patients with sepsis and severe sepsis/shock are shown in Figure 1. Among patients with intra-abdominal infection, expression of TREM -1 on neutrophils and monocytes, was significantly lower at severe sepsis/shock compared with sepsis (P = 0.009 for neutrophils, P = 0.022 for monocytes). That was also the case for TREM-1 expression on neutrophils among patients with VAP/HAP (P = 0.049 between severe sepsis/shock and sepsis).

When comparing patients at severe sepsis with patients at septic shock, it was found that expression of TREM-1 on neutrophils was further decreased in the event of septic shock within patients with intrabdominal infection (P = 0.049 compared with severe sepsis) and within patients with VAP/HAP (P = 0.009 compared with severe sepsis).

No significant correlation was found between expression of TREM-1 on neutrophils and monocytes and APACHE II score.

**Figure 2:**
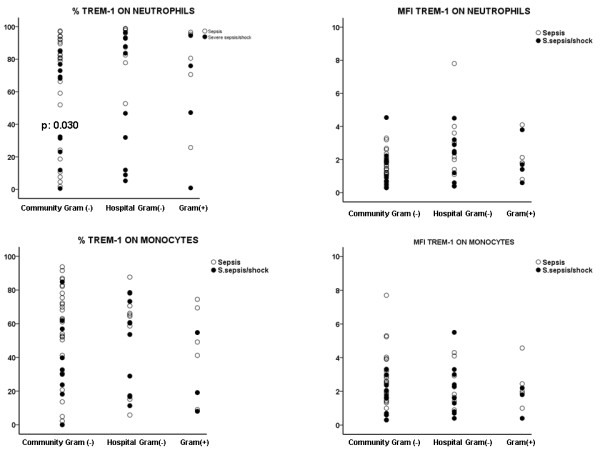
**Expression of TREM-1 on the first day of presentation of sepsis and of severe sepsis/septic shock on the surface of neutrophils and monocytes in relation with the type of the pathogen**. P values indicate statistically significant differences between sepsis and severe sepsis/shock at the specific type of pathogen.

Gram-negative community-acquired bacteria were the most frequent isolated pathogens. Expression of TREM-1 on neutrophils of patients infected by the latter type of pathogens was significantly lower in severe sepsis/shock than in sepsis (P = 0.030) (Figure 2). No differences were found between severe sepsis and septic shock. When analysis involved only patients with sepsis, the MFI of TREM-1 on neutrophils of patients bearing infections by community-acquired Gram-negative bacteria was lower than the MFI of TREM-1 on neutrophils of patients bearing infections by hospital-acquired Gram-negative bacteria (P = 0.003 between them).

**Figure 3:**
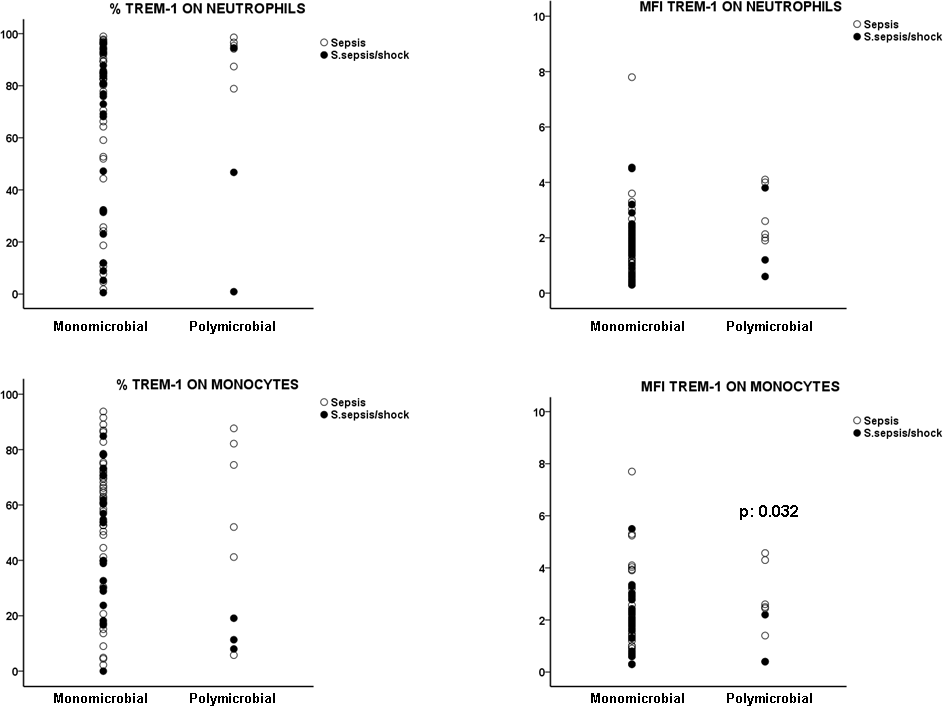
**Expression of TREM-1 on the first day of presentation of sepsis and of severe sepsis/septic shock on the surface of neutrophils and monocytes in relation with the presence of infection of polymicrobial or monomicrobial origin**. P values indicate statistically significant differences between sepsis and severe sepsis/shock at the specific cause.

Among the total patients where the causative pathogen was isolated, septic syndrome was of monomicrobial origin in 78 patients; in 20 patients septic syndrome was of polymicrobial origin. MFI of TREM-1 on the membrane of monocytes of patients bearing infections of monomicrobial origin at severe sepsis/shock was greater than of patients with infections of polymicrobial origin at severe sepsis/shock (P = 0.032) (Figure 3). No differences were found between severe sepsis and septic shock. However, analysis of the expression of TREM-1 within patients with sepsis found that it was significantly greater in patients with polymicrobial infections than in patients with monomicrobial infections (P = 0.040 regarding differences of % expression of TREM-1 on neutrophils; P = 0.008 regarding differences of MFI expression of TREM-1 on neutrophils).

**Figure 4:**
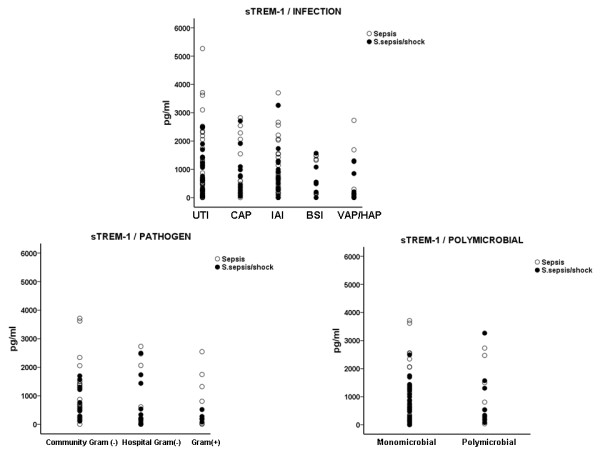
**Serum concentrations of sTREM-1 on the first day of presentation of sepsis and of severe sepsis/septic shock in relation with the causative type of infection and with the implicated pathogen**.

Differences similar to those found for the expression of TREM-1 between sepsis and severe sepsis/shock were not observed for sTREM-1 (Figure 4).

## Discussion

TREM-1 is a receptor engaged on the cell membranes of neutrophils and of monocytes that is expressed during the septic syndrome upon stimulation with microbial products. Activation of TREM-1 leads to the transcription of genes of pro-inflammatory cytokines that play a pivotal role in the amplification of the immune response. During the septic process, surface TREM-1 expression on the cells of the innate immune system is highly modulated [18-20]. Expression of TREM-1 on the surface of circulating monocytes is increased in septic shock as evidenced in a previous study of 25 patients. Expression of TREM-1 was compared with patients with systemic inflammatory response syndrome of non-infectious origin and no data were available for patients at the stages of sepsis and of severe sepsis [21]. It seems that a circulating factor in the serum of shocked patients stimulates the expression of TREM-1 [22].

Much more evidence is available for the kinetics of the soluble counterpart of TREM-1 i.e. of sTREM-1. Concentrations are much greater in serum in the case of septic shock than in the cases of sepsis or of severe sepsis [22-24]. To this end, many authors propose that sTREM-1 may be used as a surrogate marker that helps diagnosis of critically ill patients with signs of systemic inflammatory response [25,26].

The present study provides further insight in the modulation of TREM-1 expression in sepsis. Results suggest that TREM-1 expression on the membranes of neutrophils and monocytes in patients with septic syndrome is related to the type of infection. Expression is decreased within patients at severe sepsis who are suffering either from an intra-abdominal infection or from VAP/HAP compared with patients at sepsis suffering from similar infections. This is further decreased among patients at septic shock. Modulation of the immune status in sepsis by the underlying type of infection has also being shown in a recent publication of our group. Both innate and adaptive immune responses differed considerably between sepsis due to VAP and sepsis due to other types of infection. VAP was characterized by substantial decreases of CD4-lymphocytes and immunoparalysis of monocytes in contrast to other infections [27].

Modulation of the expression of TREM-1 was also affected by the type of microbial pathogen. More precisely, expression of TREM-1 was decreased when severe sepsis/shock developed in the field of infections caused by Gram-negative community-acquired bacteria.

Expression of TREM-1 on the membranes of monocytes is decreased in patients with severe sepsis/shock and polymicrobial infections compared to patients with sepsis. Explanations for these differences remain theoretical. The ligand of TREM-1 is not clearly defined. TREM-1 receptor is mainly expressed on neutrophils and monocytes after bacterial and fungal stimuli, so as to be considered one important counterpart of the innate immune response in sepsis [7,8]. The presented data suggest that TREM-1 expression on neutrophils and monocytes is decreased upon transition from sepsis to severe sepsis/shock in a fashion depending on the underlying type of infection and on the causative pathogen. Since TREM-1 is a pro-inflammatory receptor, it may be hypothesized that the observed decrease of TREM-1 expression upon aggravation to severe sepsis/shock may be a component of the immunoparalysis taking place in sepsis. At the stage of immunoparalysis, monocytes of septic patients fail to produce a similar amount of cytokines as monocytes of the non-septic host do when stimulated ex vivo [2].

It is suggested that part of TREM-1 over-expressed on cell membranes is shed in the systemic circulation in a soluble form known as sTREM-1. sTREM-1 is considered to represent an anti-inflammatory response of the host [28]. In the present study, serum kinetics of sTREM-1 did not follow changes of the expression of TREM-1 suggesting that simple shedding of the membrane receptor did not take place.

## Conclusions

The presented results show for the first time that expression of the TREM-1 receptor on the cell membranes of circulating neutrophils and monocytes early after diagnosis differs greatly between sepsis and severe sepsis/shock in relation; this is linked with the underlying infection and the causative pathogen. These results may have a major impact on therapeutics for the management of the septic patients.

## Abbreviations

APACHE: acute physiology and chronic health evaluation; CAP: community-acquired pneumonia; COPD: chronic obstructive pulmonary disease; HAP: hospital-acquired pneumonia; LPS: lipopolysaccharide; MFI: mean fluorescence intensity; SE: standard error; sTREM-1: soluble Trigerring receptor expressed on myeloid cells-1; TREM-1: Triggering receptor expressed on myeloid cells-1; VAP: ventilator-associated pneumonia.

## Competing interests

The authors declare that they have no competing interests.

## Authors' contributions

TP participated in study design and analysis of data and wrote and approved the final manuscript.

AS, IM and AP participated in study design, performed the lab job and read and approved the final manuscript.

PK, ZA, MC, ID, PD, EJGB, IK, AM, AM, SEO, MT, CT, NM, VP and IT participated in study design, enrolled patients in the study, analyzed the data and read and approved the final manuscript.

## Pre-publication history

The pre-publication history for this paper can be accessed here:

http://www.biomedcentral.com/1471-2334/11/309/prepub
